# Reversal of Middle Cerebral Artery Stenosis by Minimally Invasive Intracerebral Hematoma Evacuation

**DOI:** 10.1227/neuprac.0000000000000087

**Published:** 2024-04-05

**Authors:** Yigit Can Senol, Mona Asghariahmadabad, Alexander Haddad, Wade S. Smith, Luis E. Savastano

**Affiliations:** * Department of Neurological Surgery, University of California, San Francisco, California, USA;; ‡ Department of Neurology, University of California, San Francisco, California, USA

**Keywords:** Intraparenchymal hemorrhage, Middle cerebral artery, Stretching, Occlusion, Stenosis

## Abstract

**BACKGROUND AND IMPORTANCE:**

Acute intracerebral hematomas are known to induce significant mass effects within the brain, leading to critical complications such as cerebral midline shift, herniation, and increased intracranial pressure. The timing and efficacy of intracerebral hematoma evacuation remain subjects of ongoing debate in current literature.

**CLINICAL PRESENTATION:**

In our case report, we present a 74-year-old female patient diagnosed with basal ganglia hematoma. The resultant mass effect from the intracerebral hematoma led to middle cerebral artery (MCA) stenosis. Notably, early-stage minimally invasive hematoma evacuation was pivotal in facilitating successful revascularization of the MCA.

**CONCLUSION:**

Our case underscores the significance of prompt identification and management of MCA stenosis arising from intracerebral hematoma. Early intervention through minimally invasive hematoma evacuation proved instrumental in achieving successful MCA revascularization. These findings emphasize the critical role of timely interventions in mitigating potential complications associated with intracerebral hematoma.

**ABBREVIATIONS:**
CTA: computed tomography angiography
EVD: external ventricular drain
LVO: large vessel occlusion.

Acute intracerebral hematomas (ICHs) can generate sufficient mass effect, tissue displacement, and increased intracranial pressure to induce middle cerebral artery (MCA) occlusion. Extrinsic stenosis of the MCA resulting from a basal ganglia hematoma can be reversed by evacuation of the hematoma. Computed tomography angiography (CTA) can provide valuable insights into the vascular anatomy surrounding the hemorrhage, helping clinicians make informed decisions about the underlying pathology of hematoma. This approach allows for a more personalized treatment strategy, balancing the need to prevent hematoma expansion while preserving adequate cerebral perfusion through collateral circulation.

Acute ICHs pose a critical neurological threat, often leading to mass effects, tissue displacement, and increased intracranial pressure.^1^ Consequently, this can result in permanent morbidities and mortalities. Basal ganglia hematomas typically occur because of hypertension, and their surgical evacuation remains a subject of debate in the current literature.^2-4^ Emergency imaging techniques, such as CTA, play a crucial role in diagnosing and evaluating underlying vascular issues, active bleeding, or vessel blockages in ICHs. While previous cases in the literature have reported vessel occlusions related to mass effects,^5^ extrinsic MCA stenosis due to ICH has not been reported before. In this case report, we demonstrate the recanalization of extrinsic stenosis concurrent with intracerebral hematoma evacuation.

## CASE PRESENTATION

**FIGURE 1.**
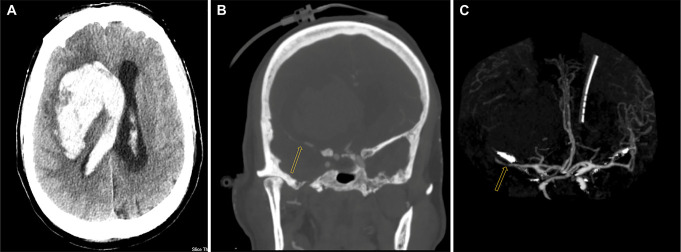
Initial CT/CTA before and after external ventricular drain insertion. **A**, The axial CT scan shows a right basal ganglia-originating hematoma extending into the ventricles with midline shifting, along with mild hydrocephalus. **B**, The CTA reveals a suspected large vessel occlusion/stenosis in the right MCA (indicated by an arrow). **C**, The CTA scan displays the hematoma and its proximity to the MCA, as well as how the hematoma and peripheral edema exert pressure on the MCA (indicated by an arrow). CT, computed tomography; CTA, CT angiography; MCA, middle cerebral artery.

A 74-year-old female patient with a history of hypertension was found down with a reduced level of consciousness by family members with a last known normal 48 hours before. The patient was intubated and emergently transported to a tertiary care center. On arrival at the emergency department, she had a Glasgow Coma Scale of 5T (E1V1TM3) with trace movement to pain on the left side and anisocoria with a dilated and nonreactive right pupil. The patient's blood pressure was measured at 160/77 mm Hg. A noncontrasted head computed tomography scan demonstrated a large intraparenchymal hematoma (measuring 7.3 × 5.2 × 4.0 cm, with an approximate volume of 76 mL) centered in the right basal ganglia and frontal lobe, resulting in ipsilateral uncal herniation and extending into the lateral, 3rd, and 4th ventricles (Figure 1A). The scan also indicated the presence of obstructive hydrocephalus, accompanied by a 12-mm right to left midline shift (Figure 1B). After a discussion with the family about the goals of care, an emergent left frontal external ventricular drain (EVD) was placed.

After EVD placement, a CTA was performed to assess for an underlying vascular abnormality or evidence of active hemorrhage. CTA demonstrated irregular narrowing of the right MCA with multifocal occlusions and lateral displacement of the M3 cortical segment or candelabra. In addition, evidence of stretching in the M1 segment significantly reduced distal MCA blood flow, and perihematomal brain edema was identified (Figure 1C).

**FIGURE 2.**
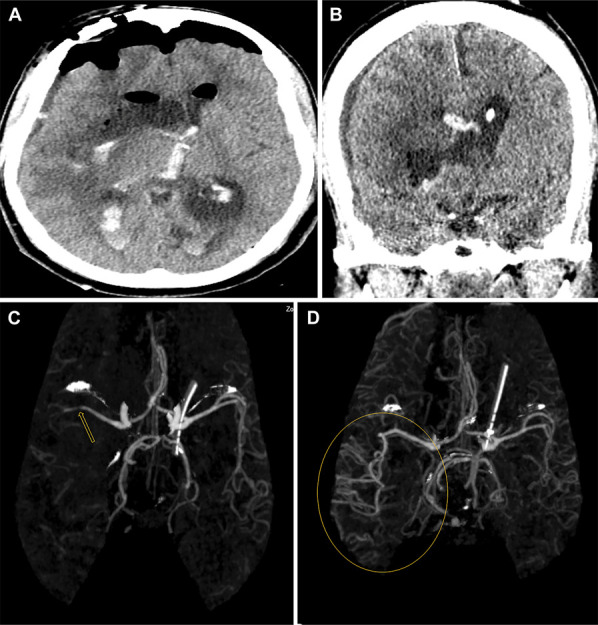
Postoperative CT and CTA scans show improvement in midline shift and recanalization of MCA after hematoma evacuation. **A**, In the early postoperative period, the axial CT scan reveals an improvement in midline shift. Despite a slight hemorrhage visible in the coronal section within the ventricle, the external ventricular drain placement is appropriate and provides effective ventricular drainage (arrow) **B**, After the postoperative endoscopic hematoma evacuation, a return to normal filling is observed in the MCA vessel **C**, and there is an improvement in blood flow in the distal regions (circle). **D**, In the preoperative CTA scan a suspicious appearance of a large vessel occlusion is observed just before the bifurcation (indicated by an arrow) of the right MCA's distal segment. There is a noticeable reduction in blood flow in the distal MCA territory. CT, computed tomography; CTA, CT angiography; MCA, middle cerebral artery.

**FIGURE 3.**
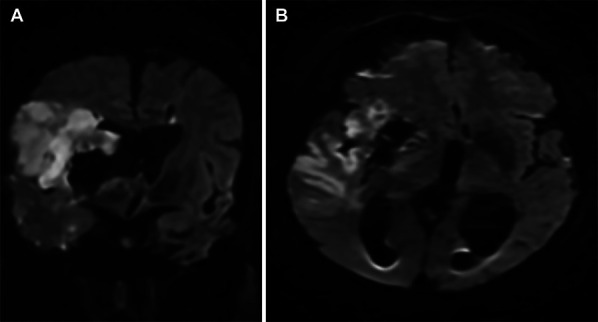
Postoperative MR imagesafter endoscopic hematoma evacuation. Both the **A**, coronal and **B**, axial diffusion weight MR scans show an infarct area in the middle cerebral artery region during the postoperative period. MR, magnetic resonance.

A decision was made to proceed with an endoscopic ICH evacuation within the first 8 hours from the first admission to emergency room. To this end, a right-sided supraorbital mini craniotomy was performed by eyebrow incision. A tubular retraction system was born into the ICH under neuronavigation, followed by endoscopic-assisted hematoma aspiration. A postoperative head computed tomography and CTA demonstrated near-complete evacuation of the hematoma with improvement of midline shift (Figure 2A and 2B) and complete recanalization of the right MCA candelabra with resolution of the stretching of the MCA (Figure 2C) comparing with preoperative CTA scan (Figure 2D). MRI obtained on postoperative day 2 revealed a restricted diffusion confined to the right MCA territory consistent with an acute ischemic stroke (Figure 3A and 3B). Workup to large vessel occlusion (LVO), including cardiac echocardiogram and imaging of the aortic arch and carotids, revealed no abnormalities.

Eight days after admission, the EVD was removed. At the last follow-up, 3 months after the presentation, the patient showed good neurological recovery with the ability to construct complete sentences, respond to yes/no questions, and follow complex commands with a modified Rankin Score(mRS) of 2.

The patient's informed consent was obtained for the publication of anonymized data.

## DISCUSSION

This illustrative case report presents a case of mechanical MCA stenosis from extrinsic compression and stretching from a large basal ganglia hematoma with recanalization after hematoma evacuation. This case provides an opportunity to discuss extrinsic MCA stenosis as an etiology of ischemic stroke and introduces an additional consideration in the management of blood pressure in ICH. Furthermore, it highlights the potential role of early hematoma evacuation (<8 hours) in limiting secondary injury.^6^

The most common cause of nonlacunar ischemic strokes are emboli derived from the heart and extracranial large arteries.^7^ Hemodynamic mechanisms, vasospasm, and intrinsic (ie, initiated in situ) thrombotic blockage are collectively less frequent contributors to ischemic stroke than emboli.^8^ The least common causes of ischemic strokes are secondary to LVO because of extrinsic factors such as tumors.^5^ In current literature, extrinsic compression of the proximal part of the MCA from an acute ICH is not a previously recognized cause of LVO. Overall, this case highlights the potential for parenchymal hematomas to cause temporary vessel stenosis and occlusions, which can be treated with and reversed with the reduction of mass effect through ICH evacuation. We would like to define this phenomenon as ICH-induced vessel stenosis, likely resulting from a combination of local mass effect with arterial displacement and stretching, direct compression from a rapidly expanding hematoma, and elevated intracranial pressure.^9^

Although direct MCA stenosis from an ICH is not described in the literature and the presence of a penumbra surrounding a hematoma analogous to ischemic stroke is a controversial topic, previous studies have demonstrated the direct compression of ICHs on surrounding vessels in humans. A previous case series involving 5 patients with basal ganglia ICH revealed that the hematoma clot within the perihemorrhagic zone could lead to vessel compression and occlusion, potentially affecting local parenchymal perfusion. Intriguingly, once the hematoma was removed, vascularization in the penumbra like rim became visible again, implicating the mass effect from an ICH in local vessel occlusion.^10^

Our findings of ICH-induced vessel stenosis add consideration when determining optimal blood pressure management in patients with acute ICH. Although exact blood pressure goals are controversial, blood pressure reduction has canonically been used to limit hematoma expansion in ICH.^4^ However, it poses a complex challenge because of the potential consequences of extrinsic compression on the surrounding vasculature by arterial occlusion and preventing collateral blood flow. A significant drop in blood pressure can potentially favor arterial occlusion, restricting blood flow to vital areas of the brain. In addition, it can hinder the flow through collateral vessels, essential for maintaining cerebral perfusion in vessel blockages. Although a drop in blood pressure would intuitively decrease the risk of hematoma expansion, increasing blood pressure may preserve collateral flow and counteract the extrinsic compression of blood vessels.^11^ Independently to the conundrum of optimal blood pressure targets in the context of a large acute ICH, we hypothesize that the benefit of very early evacuation of hematoma recently demonstrated in Early Minimally Invasive Removal of Intracerebral Hemorrhage (ENRICH) trial could be partially due to the reduction of secondary ischemic injury.^2^

## CONCLUSION

In conclusion, extrinsic stenosis of the MCA from a large acute hematoma can occur and should be considered in the workup and management of ICH. Very early evacuation of these ICHs can restore arterial patency and should be considered as an added benefit of the intervention.

## References

[1] Balami JS Buchan AM. Complications of intracerebral haemorrhage. Lancet Neurol. 2012;11(1):101-118. 22172625 10.1016/S1474-4422(11)70264-2

[2] Ratcliff JJ Hall AJ Porto E Early minimally invasive removal of intracerebral hemorrhage (ENRICH): study protocol for a multi-centered two-arm randomized adaptive trial. Front Neurol. 2023;14:1126958. 37006503 10.3389/fneur.2023.1126958 PMC10061000

[3] Butcher KS Jeerakathil T Hill M The intracerebral hemorrhage acutely decreasing arterial pressure trial. Stroke. 2013;44(3):620-626. 23391776 10.1161/STROKEAHA.111.000188

[4] Anderson CS Huang Y Wang JG Intensive blood pressure reduction in acute cerebral haemorrhage trial (INTERACT): a randomised pilot trial. Lancet Neurol. 2008;7(5):391-399. 18396107 10.1016/S1474-4422(08)70069-3

[5] Mori K Takeuchi J Ishikawa M Handa H Toyama M Yamaki T. Occlusive arteriopathy and brain tumor. J Neurosurg. 1978;49(1):22-35. 660265 10.3171/jns.1978.49.1.0022

[6] Kellner CP Schupper AJ Mocco J. Surgical evacuation of intracerebral hemorrhage: the potential importance of timing. Stroke. 2021;52(10):3391-3398. 34187180 10.1161/STROKEAHA.121.032238

[7] Campbell BCV Khatri P. Stroke. Lancet. 2020;396(10244):129-142. 32653056 10.1016/S0140-6736(20)31179-X

[8] Hart RG Diener HC Coutts SB Embolic strokes of undetermined source: the case for a new clinical construct. Lancet Neurol. 2014;13(4):429-438. 24646875 10.1016/S1474-4422(13)70310-7

[9] Han HC Chesnutt JKW Garcia JR Liu Q Wen Q. Artery buckling: new phenotypes, models, and applications. Ann Biomed Eng. 2013;41(7):1399-1410. 23192265 10.1007/s10439-012-0707-0 PMC3618579

[10] Younsi A Scherer M Unterberg AW Orakcioglu B. Visualization of pressure related vessel compression in the perihemorrhagic zone during endoscopic ICH evacuation. Clin Neurol Neurosurg. 2016;147:64-70. 27295604 10.1016/j.clineuro.2016.05.020

[11] Anadani M Orabi MY Alawieh A Blood pressure and outcome after mechanical thrombectomy with successful revascularization. Stroke. 2019;50(9):2448-2454. 31318633 10.1161/STROKEAHA.118.024687

